# Factors that determine the degree of fulfilment of expectations for entrepreneurs from the business incubator programmes

**DOI:** 10.1007/s11365-022-00818-1

**Published:** 2022-12-06

**Authors:** Arta Antonovica, Javier de Esteban Curiel, Beatriz Rodríguez Herráez

**Affiliations:** Department of Business Economics, Rey Juan Carlos University, C/Paseo de Artilleros S/N, CP-28032 Madrid, Spain

**Keywords:** Entrepreneur, Business incubator, Hard skills, Soft skills, Factor analysis, Multiple linear regression

## Abstract

The uncertain economic situation that was experienced because of the global financial crisis in 2008 and the exponential growth in the use of new technologies in different industries has caused some individuals to become entrepreneurs through the development of a variety of new skills. The main objective of this research paper is to discover what factors significantly determine the degree of fulfilment of expectations for entrepreneurs who have graduated from the incubator programme. Entrepreneurs may expect that a business incubator programme is a key element for their economic development and that it provides them with skills for good image and professional recognition. From the methodological point of view, a survey technique was conducted on 100 entrepreneurs who had already graduated from different business incubator programmes in Madrid, Spain. For the data exploration phase, an Exploratory Factor Analysis was used, which made it possible to identify 10 factors. With the Multiple Linear Regression analysis, these newly created and named factors were ordered by level of importance. The main findings show that most statistically significant factors consist of variables that are related to behaviour, attitude and hard skills (trained).In turn, results demonstrate that soft skills (innate) also play a certain role for fulfilling expectations for developing a successful company. Study confirms that continuous managerial training programmes for entrepreneurs in the twenty-first century are a substantial part for obtaining new skills, knowledge, insights, experiences, and change of behaviours and attitudes of different aspects needed for successful company leadership and management.

## Introduction

The loss of jobs (Davis & von Warcher, 2011), the incorporation of new intelligent technologies into the business world (Willcocks, 2020) and the greater competition in search for a job, has complicated access to the labour market for a significant number of individuals, especially in the private sector. All this, together with the growing economic uncertainty affected by the financial crisis in 2008 (Dom et al, 2016), the Covid-19 pandemic (Dang & Nguyen, 2021), the present war in Ukraine (International Labour Organization, 2022) and the global logistic problems (AsianaUSA, 2022), is motivating the need to seek solutions for finding and creating self-employment. In this context, public administrations and competent bodies in Spain have created a set of rules and infrastructure to help these new entrepreneurs in two ways, on the one hand, by providing training and on the other hand, by helping to implement entrepreneurial ideas.

Entrepreneurship is a key factor in the economic and social development of countries (Czarniewski, 2016). With the entrepreneurial initiatives, it is possible to transform an opportunity into a competitive advantage, which generates value for the company itself and for the local economy. The public administration is aware of the benefits that entrepreneurship brings in terms of reducing unemployment and thus, fighting poverty and the problems that it may cause, while improving living conditions of individuals. Hence, it is also necessary to consider the social impact that entrepreneurship has, because it helps individuals with the achievement of their social objectives (Nikolaev et al., 2020). That is why, it can be asserted that entrepreneurship is a multidimensional concept that has a double economic and social utility (Acs et al., 2013).

For these reasons, in recent years there have been numerous academic authors who have studied entrepreneurship and its business consequences, but this research paper is intended to analyse, whether the empowerment for entrepreneurship is learned or if it is something innate to the individual, i.e., if the skills that are considered necessary to be an active entrepreneur can be learned and worked on. To that end, this research tries to identify different factors, hard skills or technical training (working with equipment and software) and soft skills training (interpersonal or intrapersonal focus), social influence, entrepreneurial behaviour, attitudes or organisational legitimacy, in order to determine the degree of fulfilment of expectations for entrepreneurs (Williams, 2001). In this sense, Laker and Powell (2011) identified 10 inherent differences that exist between hard and soft skill training.

This research exhibits new contributions to science as we have reduced 32 entrepreneurial variables of an incubator programme into 10 new factors of success, considering the variables presented in previous studies from the Entrepreneurial Event Model and the Theory of Planned Behaviour. The research also brings new insights to the existing literature as it helps to understand the fulfilment of expectations for entrepreneurs of an incubator programme.

On basis of the previously explained context, the following research objectives have been proposed:

1. To find out if it is beneficial for the development of entrepreneurship to participate in the business incubator programmes: failure or success.
2. To operationalise variables into more manageable factors for those entrepreneurs who have graduated from the incubator programme.
3. To explore what factors significantly determine the degree of fulfilment of expectations for entrepreneurs who have graduated from the incubator programme.

To accomplish the proposed objectives, a survey technique is used with entrepreneur graduates who have been participating in the Incubator programmes in the Community of Madrid. A survey is the most appropriate quantitative research technique in social sciences for obtaining opinions and attitudes from the specific target population. The results, based on properly applied sample technique, have been statistically examined with Exploratory Factor Analysis (EFA) and Multiple Linear Regression to be projected to the entire population. Findings indicate that ten discovered mixed factors based on 32 variables, such as hard and soft skills, attitude, behaviour, social influence, organisational legitimacy and business incubator programme variables, lead to fulfilment of expectations for entrepreneurs from graduate business incubator programmes.

## Theoretical background

### Entrepreneur and its characteristics

An entrepreneur could be defined as a person who, based on an idea or an opportunity, decides to set up his or her own business. There are vocational entrepreneurs who choose this option as the beginning of their professional life, and others who come to this option due to a series of circumstances and experiences that lead up to it. In both cases, there are always certain motivations that bring them to that decision. These motivations directly influence the intention of these persons that will later manifest in their behaviour, which in many cases will make the difference between success or failure (Ardichvili et al., 2003).

Because entrepreneurs are supposed to be leaders, their characteristics influence the project´s future development. The profile of entrepreneurs is characterised by the skills, knowledge and attitudes they possess, being able to achieve some of these through acquired knowledge, while others are independent of that factor and will be the ones that allow differentiating one entrepreneur from another.

The entrepreneur must have developed skills, fundamentally those related to creativity, the ability to work in a team, flexibility, active communication, analytical skills, motivation and perseverance (World Economic Forum, 2019; Palos-Sánchez et al., 2020).

Thus, skills are innate human abilities that can be put into practice with dexterity and ease. By working on these skills and using them continuously, a total mastery of them can be achieved, which would later be referred to as competencies. Skills are related to a single proficiency and competencies go beyond encompassing skills, knowledge and abilities. A competency is stated as a capability or ability to do something and it consists of different behaviours (visible acts) and intents (invisible acts). The behaviours are alternate manifestations of the intent, as appropriate in various situations or times (Boyatzis, 2008). The Theory of Planned Behaviour (TPB) explained in the next section tries to predict a human´s intention to engage in a behaviour at a specific time and place. Chen and Chang (2010) consider competence as a 'temporary asset', which may vanish in the absence of an interactive context. Some contextual variables such as shared values, mutual trust and mutual investment may be helpful for sustaining employee competence, aligning employee competence and organisational core competence and developing employee competence as firm-specific, thus becoming a source for sustained organisational competitive advantage. For this reason, when referring to the characteristics of an entrepreneur, skills do not require to have full control over them or to be intensively exercised (Fayolle & Gailly, 2015). Hence, competencies are more based on behaviour and skills are more based on expertise. Through this study, both characteristics could be quantifiable in order to measure the items that lead to success or failure of entrepreneurs from incubator programmes.

### Study variables

Through the literature, several variables have been highlighted that affect the degree of fulfilment of the expectations of entrepreneurs, especially when studying the case of graduates from the incubator programme. To date, the literature closer to the degree of fulfilment of expectations and motivations that influence the need to start a business is predominantly based on the Entrepreneurial Event Model (Shapero & Sokol, 1982) and the Theory of Planned Behaviour (Azjen, 1991). According to these theories, entrepreneurial intention can be predicted through attitudinal variables that trigger entrepreneurial behaviour, proposing that the cognitive component of individuals influences their behaviour, including their hard skills, soft skills, attitudes, social influence, behaviour, organisational legitimacy and business incubator programme. In this way, these seven study variables for entrepreneurship have been identified and explained below.

#### Hard skills

Hard skills are identified with the technical skills of individuals. Hard skills are learned in the classroom, training and on the job. They are demonstrated through abilities such as typing, writing, math and the use of software programs (Daykin, 2018). Although hard skills are more easily taught than soft skills, there is a wide range of aspects hard skills can cover: (1) entrepreneurial competencies, (2) marketing competence, (3) business and economic competence, (4) financial competence, (5) accounting affairs competence, (6) management competence, (7) globalisation competence, (8) business law competence, (9) enterprise resource planning competence and (10) information technology competence (Chou et al., 2010). Consequently, for successful and long-lasting entrepreneurship, certain hard skills are needed, which are knowledge and skills that are achieved with academic training and are fundamentally important to develop among future entrepreneurs (Garcez et al., 2022; Yashin et al., 2018). Also, authors, such as Yashin et al. (2018), reflect that it is fundamentally important to develop hard skills among future entrepreneurs and that a businessperson cannot be successful without instrumental skills.

#### Soft skills

To achieve business success, it is necessary to have certain qualities, attitudes and social aptitudes identified as soft skills, since they make it possible to differentiate and achieve the survival of the business idea (Ibrahim et al., 2017b). Tem et al. (2020) mention that soft skills refer to all aspects of generic skills that include both cognitive elements and non-academic skills. Hence, soft skills are identified as some of the most critical skills in the current global job market, particularly in the area of entrepreneurship. Similarly, Sadq (2019) comments that the soft skills are vital skills for effective performance in the twenty-first century. Soft skills have become a new area of leadership now and in the future and they are a priority for high performance since the success of any organisation or business company depends to a large extent on these skills. Consequently, soft skills are transversal skills that have been studied for several years in relation to the skills that should be included in the training for future employees and new entrepreneurs (Cimatti, 2016; Succi & Canovi, 2020).

#### Attitudes

Attitudes have a subject matter (referred to as the object or target), which can be an object, a person, or an abstract idea. Attitudes are relevant to many disciplines, including marketing, advertising, political behaviour, and health, for example. Attitudes toward other people are studied in the domain of interpersonal liking, attitudes toward the self in the domain of self-esteem, and attitudes toward abstract ideas in the domain of values. Attitudes can be specific, or they can generalise across objects, with people holding attitudes that are either generally positive or generally negative (Hepler & Albarracín, 2013). Other aspect of attitudes is to understand, whether they change or not. The degree of attitude change depends on whether one adopts a theoretical conceptualisation of attitudes as being crystallised in memory, as in at-the-moment evaluations, or as hybrid structures. When attitudes are defined as a fixed memory, stored permanently for later retrieval when the opportunity and the need arise, change is difficult to explain. When attitudes are defined as constructed based on temporary considerations, such as the perceiver’s mood at a particular time (Schuldt et al., 2011), they are changing attitudes. Most likely, attitudes are partly memory based and partly constructed on the fly (Albarracin et al., 2005). Thus, this bidimensional character of attitudes allows for attitude stability as well as change. For studying entrepreneurial intention and behaviour, it can be said that attitudes can be brought to mind in the service of action goals, as in the case when considering a behavioural goal reminds us of what we like and dislike about the execution and outcomes of the behaviour (Albarracín & Handley, 2011; Albarracín et al., 2008). And entrepreneurial attitudes at both the personal level and social level elucidate how the entrepreneurial intention forms. These attitudes and intentions are associated with individual perception, and they are learnable (Ajzen, 2005).

#### Behaviour

The definition of entrepreneurial behaviour, the same as the basic definitions of “behaviour”, covers a broad range of understanding. Thus, Gartner et al., (1992) define entrepreneurial behaviour as the various behaviours and activities that individuals engage in when creating new organisations—and contrast them to the behaviours and activities of individuals involved in established organisations. But Gruber and MacMillan (2017) identify entrepreneurial behaviour as based on the notion that entrepreneurial behaviours are driven to a significant extent by the meanings that founders associate with their new firm-creation activities and are identity relevant. An identity-based perspective moves beyond traditional economic views, embedded in economic rationality, when seeking to understand entrepreneurial behaviour and include entrepreneurial activities that are not primarily self-oriented, but also other-oriented. Also, for Bird and Schjoedt (2017), entrepreneurial behaviour is not only self-oriented perspective, but it is the proximal outcome of the cognitions and emotions of entrepreneurial actors; it is also the proximal individual-centric cause of venture outcomes. Knowledge of entrepreneurial behaviour has value to actors—entrepreneurs–as it allows them to shape and change their behaviours for better outcomes and to venture stakeholders, such as investors, local governments, and employees, insofar as entrepreneurial outcomes meet their respective goals. Entrepreneurial behaviour eventually results in the creation of innovations, new competition, new jobs, and new revenue streams, and scholars from several disciplines.

#### Social influence

For understanding and analysing an entrepreneur behaviour, it is essential to study the personality traits and the social influence to which this person is subjected, as Ajzen (1991) concluded in his theory of Planned Behaviour, where he studied entrepreneurial behaviour from an approach where personal and social aspects are aligned (Díez-Martín et al., 2021). During the last three decades, sociological theories of entrepreneurship have increasingly related entrepreneurship rates to differential exposure to interpersonal influences. In particular, the review of these theories suggests that social influence drives entrepreneurial behaviour in two distinct ways: by transferring information about entrepreneurial opportunities and by enhancing an individual’s motivation to become an entrepreneur (Kacperczyk, 2013). Also, there are studies that say that the transmission of entrepreneurial values and preferences has been documented by studies showing that children of entrepreneurial parents are more likely to become involved in entrepreneurship themselves (Aldrich et al., 1998; Halaby, 2003). In this sense, Kacperczyk (2013) states that individuals exposed to entrepreneurially inclined co-workers and neighbours tend to acquire tacit knowledge and information about lucrative entrepreneurial opportunities and that they become acquainted with norms that foster entrepreneurial transition. One final remark to be made in this section is that influence could also be harder for migrant entrepreneurship with less professional networks in the labour market (Carbonell et al., 2014).

#### Organisational legitimacy

In recent decades, organisational legitimacy has received a great deal of attention from researchers in order to find a common concept for it and how it could be measured. This reflects the complexity about understanding this phenomenon (Moreno et al., 2018). However, some authors try to find a definition by expressing that organisational legitimacy is the perceived appropriateness of an organisation to a social system in terms of rules, values, norms, and definitions (Deephouse et al., 2017). It is also important to mention that legitimacy matters because it has consequences for organisations, and it has a clear effect on social and economic exchange: most stakeholders will only engage with legitimate organisations. In other words, no matter what components of the marketing mix illegitimate organisations might offer, a large number of stakeholders will not transact with entities that are regarded as illegitimate (and indeed, many stakeholders may actively avoid debated organisations as well). Thus, legitimacy affects market access (Brown, 1998; Deephouse & Carter, 2005; Pfeffer & Salancik, 1978). Also, Zimmerman and Zeitz (2002) affirm that legitimacy is an important phenomenon for new ventures because it can be used strategically to increase resources and achieve growth. A better understanding of how a new venture can acquire, build, and use legitimacy may enable it not only to overcome the liability of newness but also to grow and become an established venture. But Aldrich and Fiol (1994) summarise that generating and sustaining trusting relationships are at the heart of overcoming low legitimacy.

#### Business incubator programme

A business incubator refers to a company that assists start-ups and new companies to develop through offering services such as office space or management training (Rauch & Hulsink, 2015; Siemieniuk, 2016; Krpalek & Krpálková, 2016). They differ from technology and research parks in their devotion to early stage and start-up companies, as well as through the services/facilities they are providing. Because start-up companies lack networks, experience, and resources, business incubators provide services that assist these companies to get through the initial hurdles that they are likely to encounter during the business start-up process (Alpenidze et al., 2019). Thus, there are more business incubators that belong to the municipalities, but they collaborate with the surrounding universities and companies to integrate new entrepreneurial initiatives into society (Bennett et al., 2017; Hassan, 2020). In continuation, public business incubators are services placed at the disposal of original, generally newly created projects, to which physical accompaniment, supervision and location are offered at prices below market value. They have as their aim to help set in motion and consolidate these firms during the stages in which they are weaker. The ultimate goal consists of favouring the generation of innovative firms, inducers of high-quality jobs, which can diversify the local business fabric, thus becoming a key tool in local development (Sentana et al., 2017). Also, one of the fundamental functions of business incubators is training in order to help develop the necessary skills for each case (Salinas & Osorio, 2012). Thus, in 2008, the first business incubators emerged in Madrid (Madrid Emprende, 2022), which in addition to providing a physical space and services to entrepreneurs, also help them to grow until they are self-sustaining.

### Research model

**Fig. 1:**
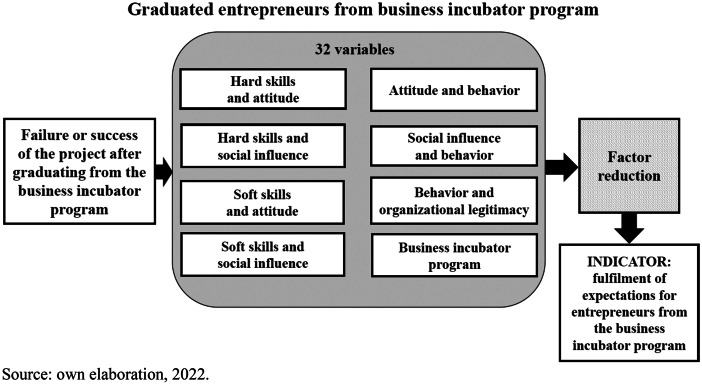
Research model of this study

In this context, Fig. 1 outlines the research model of this study with the relationship of the proposed objectives earlier on. Entrepreneurs may expect that a business incubator programme is a key element for their economic development, enhancing their skills for good image and professional recognition. Entrepreneurs may expect that business incubator programmes are flexible, efficient, and creative. With the application of a survey technique, it will be confirmed if it is beneficial (failure or success) for development of the entrepreneurial project to participate in the incubator programme. Lately, to discover determinant factors that fulfil expectations for entrepreneurs, the Exploratory Factor Analysis (EFA) has been applied that helps to optimise and reduce 32 variables in more manageable number of factors (Siddiqui et al., 2021). These 32 variables correspond to scale statements categorised in 8 groups from the 7 study variables mentioned above (hard skills, soft skills, attitudes, social influence, behaviour, organisational legitimacy, and business incubator programme). The study model and questionnaire variables are based on different scientific research related to entrepreneurial studies. The measurement of “attitude”, “social influence” and “behaviour” concerned with entrepreneurial activities is based on the papers of Fernández-Pérez et al. (2014) and Fernández-Pérez et al. (2019). The subset of “hard skills” for entrepreneurship is based on the article of Garcez et al. (2022), whereas “soft skills” is based on Tem et al. (2020). The section of “organisational legitimacy” in the entrepreneurship context is connected with the analysis of Deephouse et al. (2017). Finally, the block of questions about “business incubator” has been added by the authors ad hoc to the central focus of this groundwork.

Multiple Linear Regression analysis has then been implemented, the aim of which is to support the creation of an indicator based on statistical measurement by presenting the most to the least significant factors. These new factors from this study explain the fulfilment of expectations for entrepreneurs from the business incubator programme. They can also give wider understanding for entrepreneurship skills´ teaching and training on the academic and practical level for existing and future entrepreneurs of the twenty-first century. The results thus obtained can help other researchers as a paradigm to study new factors, for understanding necessary skills for entrepreneurship or to apply the discovered factors for examining other entrepreneurial situations.

## Methodology

### Data collection and online survey procedure

The information-gathering technique applied for this research has been an online survey (“encuestas.com” platform). In this sense, information has been collected from the people responsible for the entrepreneurs who have participated in the Incubator Programme at the Madrid offices (Vicálvaro, Carabanchel, Moratalaz, Puente de Vallecas and Móstoles). This Incubator Programme is part of the strategy from the Economic Development Agency of the Madrid City Council -Madrid Emprende- which plans to develop the business fabric of the city, as a basis for achieving sustainable economic growth that contributes to improving the well-being of the citizens of Madrid. This programme covers a period of three years, where entrepreneurs are provided with a centre for business project incubators in a work environment and the necessary support and advice to turn their ideas into feasible business plans and real companies (Madrid Emprende, 2022).

Then, so far, 452 entrepreneurs have been contacted that have participated in this Business Incubator programme since 2012 and have already graduated. Graduation is achieved after a three-year period under the programme, so at the time of this research, some have completed it and some have companies still running, while others have not made progress or have companies which are no longer active.

Given these specific characteristics (identified subjects, but difficult accessibility), the authors have decided to conduct an online survey due to its flexibility in contacting these entrepreneurs. Online survey can be used for descriptive, explanatory, and exploratory purposes. In this sense, for this research paper, it has been used for the exploratory purpose. Additionally, online surveys are mainly used in studies that have individual people as units of analysis. Surveys are excellent techniques for measuring attitudes and perceptions (Babbie, 2014).

The 452 entrepreneurs were listed in the database of the Madrid Incubator Programme with their telephone number and email address. First off, the authors sent an email on 8^th^ April 2022 with the link of the survey to be completed (self-administered). A reminder email has been sent on 18^th^ April, and a follow-up telephone call was carried out in the week of 25^th^ April and so on for those that have not replied or replied but have not submitted the survey. The last survey was completed on 12^th^ May to obtain 100 questionnaires filled out and validated. Of the total contact, 88 subjects have declined to participate and 264 have not replied (non-active email address or lack of motivation to participate).

The questionnaire was divided into five research blocks:

1. General data about each entrepreneur and his or her project: year of graduation from the Incubator programme, project implementation, initial year of the company, sector of activity, number of employees, social capital as type of company).
2. Factors for entrepreneurship: training factors (hard skills), systemic factors (soft skills), attitude, social influence, entrepreneurial behaviour, organisational legitimacy, and incubator programme.
3. Exogenous factors: effect of Covid pandemic, economic crisis, changes in legislation and technological changes.
4. Current situation of the project: project has gone ahead, company is active or has ceased, and expectations in order to create an indicator to explore if there is a quantifiable relationship between the hard and soft skills of an entrepreneur, the attitudes acquired in a business incubator, the social influence and the fulfilment of expectations once the business project has been launched.
5. Sociodemographic details of each entrepreneur: gender, age, educational level.

In order to confirm the questionnaire´s consistency, three steps have been carried out. First, from the theoretical construct (wording, variables and scale items) it was tested by the Spanish market research company “Deskmind Research (2022)” experts (a well-known team of professionals with more than 25 years of experience in market, social and opinion research). Secondly, from the methodological perspective, a pre-test of the survey was applied during the last two weeks of March 2022 by randomly sending emails with the questionnaire to 15 entrepreneurs from the Madrid Incubator Programme. Thirdly, from the statistical viewpoint, to determine the reliability and validity of the 32-item scale for the selected questionnaire, Table 1 shows that the Cronbach's Alpha used to determine the internal consistency was 0.905, which is an acceptable range for social science research.

**Table 1:** Cronbach`s Alpha for

**Reliability statistics**	
**Cronbach’s Alpha**	**N of items**
0.905	32

Source: own elaboration, 2022

In the case of scales of perceptions and predictive analysis, the greater the number of answers in the items, usually results in greater reliability in the whole scale. Hence, a 1–10 scale (compare with 1–5) for rating different statements of the questionnaire seems appropriate and has been applied for this web-based survey. Studies, such as Schmidt et at. (2013), about directors of entrepreneurship programmes also applied a survey with a 1–10 scale items. Respondents generally do not read the text anchors and go through the survey quickly, so people know habitually that a rating of 1 is the lowest score and 10 the highest. Moreover, using an even-numbered scale of 1–10 (with 10 options) does not provide a mid-point at 5, avoiding the possibility that the respondents may evade choosing this mid-point denoting indecision.

### Data analysis

With the database obtained from the incubator programme, the information has been analysed using SPSS software. Firstly, a descriptive statistical tabulation of the cases (means and percentages) has been made using the different possibilities of fulfilling the expectations of the project as table header.

Secondly, an Exploratory Factor Analysis (EFA) was performed with the list of hard and soft skills/attitudes/behaviour/social influence/legitimacy/incubator programme. A 10-factor model with Varimax rotation has been proposed. (Covariance between factors = 0). The implementation of an Exploratory Factor Analysis (EFA) was based on the expectation that all 32 variables of this questionnaire could be included under entrepreneurship. In particular, with the EFA, 10 common factors with different descriptive categories were explored which help to understand the skills for being a successful entrepreneur. Thus, by following EFA analysis rules, new factor naming was applied. Factors are typically named by considering what their most salient manifest variables have in common. Both pattern and structure coefficients should be used for this purpose, but structure coefficients may be more useful because they reflect factor-variable correlations without the confounding effect of other factors. To reduce the possibility of confusion, factors should not be given the same names as manifest variables (Watkins, 2018). Also, Tabachnick and Fidell (2007) mention that one of the challenges of this technique is that naming the factors can be problematic. Factor names may not accurately reflect the variables within the factor. Some variables are difficult to interpret because they may load onto more than one factor, which is known as split loadings. These variables may correlate with each another to produce a factor despite having little underlying meaning for the factor.

Given that it has never been explored before that way, EFA has been chosen instead of Confirmatory Factor Analysis (CFA) for its validity and reliability. Afterwards, the validity of the new factors has been evaluated through Bartlett's sphericity test, where p-value < 0.05 indicates that the matrix is adequate due to the high correlations between the variables; and the reliability with the KMO (Kaiser-Mayer-Olkin) test, where a value of at least 0.6 indicates that the partial correlations between variables are acceptable.

Next, the possible correlation between the descriptive variables (factors) and the fulfilment of the entrepreneur's expectations has been analysed.

Finally, a Multiple Linear Regression has been carried out, taking as dependent variable the degree of fulfilment of expectations, and as independent variables the entrepreneur's characteristics defined through factor analysis, obtaining a solvent model for estimating the degree of fulfilment of expectations from the hard and soft skills/ attitudes/ behaviour/ social influence/ legitimacy/ incubator programme of the entrepreneur.

Since the factors are independent of each other, it has been possible to use the standardised Beta coefficients to estimate the weight of each characteristic (factor) on the level of success/failure of the entrepreneurship.

## Results

### Descriptive statistical analysis

A descriptive statistical tabulation (Table 2) has been used to provide the basic features with the different possibilities of fulfilling the expectations of an entrepreneur project as table header, including an average indicator (0–200). Thus, 10% of the respondents who have graduated from the business incubator programme have not succeeded with the entrepreneurship project.

**Table 2:** Descriptive statistics about the different possibilities of fulfilling the expectations of an entrepreneur project

		**Which of these situations best suits your entrepreneurial activity?**			
**3.- Fulfilment of expectations**	TOTAL	It hasn’t gone ahead. It is no longer active	Still active, but below expectations	Still active, as expected	Still active, exceeds expectations
**Base: cases**	**100**	**10**	**31**	**27**	**32**
**Thinking about the current situation of your project, …** **Which of these situations best suits your case?**					
It hasn’t gone ahead. It is no longer active	**10%**	**100%**	0%	0%	0%
Still active, but below expectations	**31%**	0%	**100%**	0%	0%
Still active, as expected	**27%**	0%	0%	**100%**	0%
Still active, exceeds expectations	**32%**	0%	0%	0%	**100%**
**Fulfilment of expectations** **(0 = "It did not go ahead" to 200 "It exceeded what was expected by 100%")**					
Average fulfilment of expectations (0–200)	**100.9**	-	56.5	100.0	**176.3**

Source: own elaboration, 2022

### Exploratory Factorial Analysis

As mentioned before, Exploratory Factorial Analysis has been applied for this research, so as to discover factors empirically agreeing with Salvador and Gargallo (2006). In order to confirm the validity of the data obtained, Bartlett's sphericity test was used, where 32 items were reduced to a fewer number of dimensions. Thus, KMO was also applied to measure adequacy of the sample. In this context, Bartlett's sphericity test (p-value < 0.000) and the KMO index (0.68 higher than 0.6) justify the application of factor analysis (Table2). Both test results show the adequacy for the factorial analysis in accordance with Hair et al. (2013) and Levy and Varela (2003) (Table 3).

**Table 3:** Bartlett's sphericity test and the KMO index of this research

KMO and Bartlett's sphericity tests		
Kaiser–Meyer–Olkin measure of sampling adequacy		0.68
Bartlett's test of sphericity	Approx. Chi-squared	2499.618
	gl	496
	p-value	0.000

Source: own elaboration, 2022

Communalities indicate the amount of variance in each variable that is accounted for. In this EFA, all 32 variables are higher than 0.5 indicating a good explanation capacity of this model. At the same time variables are grouped in relation to the studied determinants explained in the theoretical framework (Table 4).

**Table 4:** Communalities of this EFA

**Communalities**		
	**Initial**	**Extraction**
VARIABLE GROUP: A- HARD SKILLS AND ATTITUDE		
Q1A1-My university studies directly influenced my interest in entrepreneurship	1	0.809
Q1A2-The training I acquired gave me insights to create my company	1	0.813
Q1A3-Training through seminars, conferences or courses helped me obtain information to create my company	1	0.807
VARIABLE GROUP: B- HARD SKILLS AND SOCIAL INFLUENCE		
Q1B1-I chose my university studies on the grounds of family tradition	1	0.707
Q1B2-I was instructing myself by participating in courses attended by my friends	1	0.750
VARIABLE GROUP: C- SOFT SKILLS AND ATTITUDE		
Q1C1-I felt able to make decisions under uncertainty	1	0.757
Q1C2-I felt able to modify decisions if conditions changed	1	0.822
Q1C3-I felt able to recognise the potential of an idea or opportunity	1	0.830
Q1C4-I felt able to discover new ways to improve existing products or services	1	0.747
Q1C5-I felt capable of creating products or services to cover unsatisfied needs of consumers	1	0.807
Q1C6-I felt capable of leading a work group	1	0.863
VARIABLE GROUP: D- SOFT SKILLS AND SOCIAL INFLUENCE		
Q1D1-I felt capable of negotiating and convincing in the relationship with other workmates	1	0.791
VARIABLE GROUP: E- ATTITUDE AND BEHAVIOUR		
Q1E1-I have always been interested in establishing my own company	1	0.843
Q1E2-I started my business proposal as soon as I had the opportunity	1	0.871
Q1E3-Being an entrepreneur brings me more advantages than disadvantages	1	0.810
Q1E4-Entrepreneurship has always been attractive to me	1	0.785
Q1E5-Being an entrepreneur gives me great satisfaction	1	0.855
Q1E6-Among the professional options I had, my favourite was to be an entrepreneur	1	0.807
Q1E7-Creating the company and maintaining it has been easy for me	1	0.685
Q1E8-Becoming an entrepreneur has been an important part of my identity	1	0.858
Q1E9-When I think about it, the term "entrepreneur" suits me	1	0.782
Q1E10-As much as possible, I avoid failure as it can damage my reputation	1	0.798
Q1E11-Failure gives us the opportunity to reflect and innovate	1	0.899
Q1E12-Many failures lead to long-term positive results	1	0.776
Q1E13-I try to avoid failure since it frustrates me	1	0.755
VARIABLE GROUP: F- SOCIAL INFLUENCE AND BEHAVIOUR		
Q1F1-My friends approve of my decision to start a business	1	0.820
Q1F2-My close family encouraged me to create a company	1	0.748
VARIABLE GROUP: G- BEHAVIOUR AND ORGANISATIONAL LEGITIMACY		
Q1G1-All the activities carried out by my organisation help to achieve the objectives	1	0.692
Q1G2-All the activities carried out by my organisation "should be done" regardless of their usefulness to achieve the objectives	1	0.679
Q1G3-My organisation develops activities which help to simplify decision-making processes, making better and more rational decisions	1	0.847
VARIABLE GROUP: H- BUSINESS INCUBATOR PROGRAMME		
Q1H1-Belonging to the business incubator helped me recognise business opportunities	1	0.705
Q1H2-Belonging to the business incubator helped me create my company	1	0.817
*Extraction method: principal component analysis*		

Source: own elaboration, 2022

Several iterations have been taken to come up with the optimal number of factors. In particular, total variance explained by 5 factors/components is 79.179% showing the data is useful (Table 5).

**Table 5:** Total variance explained by factors analysis

Total variance explained									
**Component**	**Initial eigenvalues**			**Extraction eigenvalues**			**Rotation eigenvalues**		
	Total	% of variance	Cumulative %	Total	% of variance	Cumulative %	Total	% of variance	Cumulative %
1	10.044	31.388	31.388	10.044	31.388	31.388	6.516	20.363	20.363
2	3.404	10.638	42.027	3.404	10.638	42.027	3.763	11.759	32.122
3	2.65	8.282	50.309	2.65	8.282	50.309	2.525	7.892	40.014
4	1.858	5.805	56.114	1.858	5.805	56.114	2.252	7.039	47.053
5	1.701	5.315	61.429	1.701	5.315	61.429	2.14	6.689	53.742
6	1.524	4.761	66.19	1.524	4.761	66.19	2.073	6.478	60.22
7	1.168	3.651	69.842	1.168	3.651	69.842	1.724	5.386	65.607
8	1.1	3.437	73.278	1.1	3.437	73.278	1.638	5.12	70.726
9	0.968	3.025	76.303	0.968	3.025	76.303	1.492	4.663	75.389
10	0.92	2.876	79.179	0.92	2.876	79.179	1.213	3.79	79.179

Source: own elaboration, 2022

Sequentially, to obtain an optimal number of factors, various steps were taken: (1) identification of the variables, which correlations are the most elevated in absolute terms, and (2) assignation of a representative name of the created factors on the basis of the variables that they include.

Thus, in order to interpret the factors and reduce the data correctly, factor rotation must be performed. In this research paper, the rotated component matrix had been carried out, which tries to minimise the number of variables with high loads in a factor, additionally, the total explained variance or the communality of each variable cannot be altered.

Table 6 presents the rotated component matrix of the studied variables that determines what each created component represents. In this study, 10 factors have been identified.

**Table 6:** Rotated component matrix

Rotated component matrix	Component
**Factor 1**	
Q1E2-I started my business proposal as soon as I had the opportunity	0.860
Q1E1-I have always been interested in establishing my own company	0.851
Q1E8-Becoming an entrepreneur has been an important part of my identity	0.820
Q1E5-Being an entrepreneur gives me great satisfaction	0.811
Q1E4-Entrepreneurship has always been attractive to me	0.792
Q1E9-When I think about it, the term "entrepreneur" suits me	0.772
Q1E6-Among the professional options I had, my favourite was to be an entrepreneur	0.695
Q1E3-Being an entrepreneur brings me more advantages than disadvantages	0.584
Q1E12-Many failures lead to long-term positive results	0.499
Q1G3-My organisation develops activities to help simplify decision-making processes, making better and more rational decisions	0.448
Q1E11-Failure gives us the opportunity to reflect and innovate	0.444
Q1E7-Creating the company and maintaining it has been easy for me	0.373
**Factor 2**	
Q1C3-I felt able to recognise the potential of an idea or opportunity	0.829
Q1C2-I felt able to modify decisions if conditions changed	0.761
Q1C4-I felt able to discover new ways to improve existing products or services	0.743
Q1C1-I felt able to make decisions under uncertainty	0.729
Q1C5-I felt capable of creating products or services to cover unsatisfied needs of consumers	0.615
Q1C6-I felt capable of leading a work group	0.485
**Factor 3**	
Q1H2-Belonging to the business incubator helped me create my company	0.870
Q1H1-Belonging to the business incubator helped me recognise business opportunities	0.739
Q1F1-My friends approve of my decision to start a business	0.551
Q1A3-Training through seminars, conferences or courses helped me obtain information to create my company	0.422
**Factor 4**	
Q1A1-My university studies directly influenced my interest in entrepreneurship	0.882
Q1A2-The training I acquired gave me insights to create my company	0.835
Q1A3-Training through seminars, conferences or courses helped me obtain information to create my company	0.536
**Factor 5**	
Q1B2-I was instructing myself by participating in courses attended by my friends	0.793
Q1B1-I chose my university studies on the grounds of family tradition	0.703
Q1A3-Training through seminars, conferences or courses helped me obtain information to create my company	0.436
Q1F2-My close family encouraged me to create a company	-0.459
**Factor 6**	
Q1E10-As much as possible, I avoid failure as it can damage my reputation	0.791
Q1E13-I try to avoid failure since it frustrates me	0.771
Q1F2-My close family encouraged me to create a company	0.521
Q1F1-My friends approve of my decision to start a business	0.378
**Factor 7**	
Q1E11-Failure gives us the opportunity to reflect and innovate	0.774
Q1E12-Many failures lead to long-term positive results	0.588
Q1G1-All the activities carried out by my organisation help to achieve the objectives	0.558
**Factor 8**	
Q1D1-I felt capable of negotiating and convincing in the relationship with other workmates	0.837
Q1C6-I felt capable of leading a work group	0.556
Q1C5-I felt capable of creating products or services to cover unsatisfied needs of consumers	0.412
**Factor 9**	
Q1G3-My organisation develops activities to help simplify decision-making processes, making better and more rational decisions	0.724
Q1E7-Creating the company and maintaining it has been easy for me	0.550
Q1G1-All the activities carried out by my organisation help to achieve the objectives	0.362
Q1C6-I felt capable of leading a work group	0.339
**Factor 10**	
Q1G2-All the activities carried out by my organisation "should be done" regardless of their usefulness to achieve the objectives	0.630

Source: own elaboration, 2022

Once the 10 factors were identified from the rotated component matrix, they were named (Table 7) taking into account the statements with common aspects and, at the same time, the components that have the highest obtained value. As it is mentioned in the scientific literature and explained in methodology, naming includes some degree of the researcher’s personal interpretation, which can be biased, because factor names may not accurately reflect the variables within the factor. In this sense, when naming factors for this study, interpreted statement variables were included in the factor by adding adjective/ characterisation to the concept “entrepreneur”. Moreover, each newly named factor was classified by adding the variable group to which it belongs, considering the statements integrated in Table 4.

**Table 7:** New factor naming

Factors	New factor naming	Variable types
Factor 1	Entrepreneur by nature	Attitude and behaviour (11 variables) Behaviour and organisational legitimacy (1 variable)
Factor 2	Intuitive entrepreneur	Soft skills and attitude (6 variables)
Factor 3	Group influence entrepreneur	Business incubator programme (2 variables) Social influence and behaviour (1 variable) Hard skills and attitude (1 variable)
Factor 4	Educated entrepreneur	Hard skills and attitude (3 variables)
Factor 5	Social influence entrepreneur	Hard skills and social influence (2 variables) Hard skills and attitude (1 variable) Social influence and behaviour (1 variable)
Factor 6	Social-perfectionist entrepreneur	Attitude and behaviour (2 variables) Social influence and behaviour (2 variable)
Factor 7	Optimistic entrepreneur	Attitude and behaviour (2 variables) Behaviour and organisational legitimacy (1 variable)
Factor 8	Self- confident entrepreneur	Soft skills and social influence (1 variable) Soft skills and attitude (2 variables)
Factor 9	Rational entrepreneur	Behaviour and organisational legitimacy (2 variables) Attitude and behaviour (1 variable) Soft skills and attitude (1 variable)
Factor 10	Adventurous entrepreneur	Behaviour and organisational legitimacy (1 variable)

Source: own elaboration, 2022

Thus, Factor 1 “Entrepreneur by nature” consists of 12 statements, which include eleven “attitude and behaviour” variables and one “behaviour and organisational legitimacy” variable. This newly created factor is related to the idea that, to be an entrepreneur, this concept must always be on their mind and they must have passion for creating their own company. Factor 2 “Intuitive entrepreneur” includes 6 “soft skill and attitude” statements, which relate to recognition and feeling of new opportunities. Factor 3 “Group influence entrepreneur” contains 4 variables (two belong to the “business incubator programme”, one group to “social influence and behaviour” and one to “hard skill and attitude variable). The biggest importance of this factor, include statements where different groups, such as business incubators and friends encourage the person to be an entrepreneur. Factor 4 “Educated entrepreneur” has 3 components (all three belong to “hard skills and attitude” variable group), and they present statements, which express the idea that entrepreneurial training at university level or other courses inspired the person to create a company. Factor 5 “Social influence entrepreneur” consists of 4 components (two belong to “hard skills and social influence” variable group, one to “hard skills and attitude” and one to “social influence and behaviour”). The greatest importance for naming this factor included statements linked to close friends and family influence on the study and training choices or the decision to establish a business company. Thus, factor 6 “Social-perfectionist entrepreneur” has 4 statements where two belong to the “attitude and behaviour” variable group and the other two with lower statistical values fit into the “social influence and behaviour” group. In this context, the first two variables, on one hand, present the idea of avoidance of failures and on the other hand, the last two variables of this factor talk about approval of friends/family for doing well in business. Factor 7 “Optimistic entrepreneur” involves 3 components (two “attitude and behaviour” and one “behaviour and organisational legitimacy” variables), which indicate that failures are entrepreneurial experiences that give opportunities in the long run. Factor 8 “Self- confident entrepreneur” consists of 3 components, which reveal that these entrepreneurs have self-confidence for group leadership and market opportunities, and all three statements belong to the “soft skills and social influence or attitude” variable groups. Factor 9 “Rational entrepreneur” consists of 4 components where the biggest influence is founded on rationality-based decisions for managing a business company and its processes. Hence, this factor variables belong to the following groups: “behaviour and organisational legitimacy” two variables, “attitude and behaviour” one variable and “soft skills and attitude” also one variable. The last one, factor 10 “Adventurous entrepreneur” has only one component (“behaviour and organisational legitimacy” variable), where decisions affected by the risks are considered as business advantage.

### Correlation between the descriptive variables (factors) and the fulfilment of the entrepreneur's expectations

As dependent variable, the authors have selected the item “Fulfilment of expectations” (0 = "It did not go ahead" to 200 "It exceeded what was expected by 100%") for further measurement of the model. Table 8 provides a simple descriptive summary about the measures of the dependent variable.

**Table 8:** Descriptive statistics of the dependent variable: “Fulfilment of expectations”

Descriptive statistics	
N	100
Mean	100.9
Standard error of the mean	6.17685
Median	100
Mode	100
Standard deviation	61.76847
Variance	3815.343
Asymmetry	0.143
Skewness standard error	0.241
Kurtosis	-0.901
Kurtosis standard error	0.478
Range	200
Minimum	0
Maximum	200
Sum	10,090

Source: own elaboration, 2022

To categorise and rank the dependent variable “Fulfilment of expectations” (0 = "It did not go ahead" to 200 "It exceeded what was expected by 100%"), it has been classified by intervals (Table 9).

**Table 9:** Interval data of the dependent variable: “Fulfilment of expectations”

**Intervals**	**N**		
It hasn’t gone ahead. It is no longer active	10	10	It hasn’t gone ahead. It is no longer active
Only fulfil between 15 and 50% of expectations	10	31	Still active, but below expectations
Only fulfil between 51 and 99% of expectations	21		
Fulfilled expectations (100%)	27	27	Still active, as expected
Exceeded expectations between 1 and 50%	6	32	Still active, exceed expectations
Exceeded expectations between 51 and 100%	15		
Exceeded expectations more than 100%	11		
Total:	100	100	

Source: own elaboration, 2022

With regards to the independent variables, which are the factors obtained above, Table 10 shows the descriptive statistics in an informative way.

**Table 10:** Descriptive statistics of the independent variables: factors

Factors	N	Min *	Max *	Mean *	Std. Deviation
FACTOR 1-Entrepreneur by nature	100	0.0	9.0	4.6	2.5
FACTOR 2-Intuitive entrepreneur	100	4.0	10.0	7.9	1.4
FACTOR 3-Group influence entrepreneur	100	0.0	8.9	4.4	2.6
FACTOR 4-Educated entrepreneur	100	0.0	10.0	5.5	2.9
FACTOR 5-Social influence entrepreneur	100	0.0	9.7	0.9	1.9
FACTOR 6-Social-perfectionist entrepreneur	100	0.0	10.0	5.7	2.7
FACTOR 7-Optimistic entrepreneur	100	0.0	7.4	3.7	2.1
FACTOR 8-Self- confident entrepreneur	100	0.6	10.0	5.6	2.2
FACTOR 9-Rational entrepreneur	100	0.0	8.1	3.1	1.9
FACTOR 10-Adventurous entrepreneur	100	0.0	7.1	2.6	1.9

*Theoretical scale: 0 "Not applicable at all" a 10 "Totally applicable"

Source: own elaboration, 2022

Then, the correlation between the dependent variable “Fulfilment of expectations” and the independent variables (10 obtained factors) was calculated to figure out the extent to which these two variables are linearly related (Table 11). “FACTOR 2—Intuitive entrepreneur”, “FACTOR 6—Social-perfectionist entrepreneur” and “FACTOR 9—Rational entrepreneur” have the highest relationships.

**Table 11:** Correlation between the dependent and independent variables

Factors	Pearson's	Sig
	**Correlation**	**(Bilateral)**
FACTOR 1-Entrepreneur by nature	0.132	0.041
FACTOR 2-Intuitive entrepreneur	0.290	0.003
FACTOR 3-Group influence entrepreneur	0.085	0.399
FACTOR 4-Educated entrepreneur	0.070	0.488
FACTOR 5-Social influence entrepreneur	0.129	0.201
FACTOR 6-Social-perfectionist entrepreneur	0.291	0.003
FACTOR 7-Optimistic entrepreneur	0.084	0.409
FACTOR 8-Self- confident entrepreneur	0.128	0.204
FACTOR 9-Rational entrepreneur	0.249	0.012
FACTOR 10-Adventurous entrepreneur	0.146	0.149

Source: own elaboration, 2022

### Multiple linear regression

After the correlation analysis, a Multiple Linear Regression was conducted in order to propose an indicator that determines the degree of fulfilment of expectations based on the hard and soft skills, including attitude, social influence, the legitimacy of an incubator programme declared by the entrepreneur of an incubator programme and configured in the 10-factors´ model. With an R of 0.813 and an adjusted R square of 0.569, it can be considered that the obtained model in Fig. 1 is reliable (Table 12).

**Table 12:** Summary of the model

Model	R	R-squared	Adjusted R-squared	Estimation standard error
1	,813a	0.569	0.569	32.00081

a Predictors: (Constant), FACTORS 1 to 10

b Dependent variable: Fulfilment of expectations (0 = "It did not go ahead" to 200 "It exceeded what was expected by 100%")

Source: own elaboration, 2022

Since the factors are independent of each other, the standard coefficients´ Beta can be used to estimate the weight of each characteristic (factor) concerning the degree of fulfilment of entrepreneur expectations (Table 13). In this sense, considering the hard and soft skills, attitudes, social influence, behaviour, organisational legitimacy and business incubator programme interpretation in relation to newly labelled factors in Table 7, the importance of transformation in % of the factor weight can be determined.

**Table 13:** Coefficients of the model

**Factor**	**Non-standard coefficients**		**Standard coefficients**				
	**B**	**Standard error**	**Beta**	**t**	**Sig**	**Beta **^**2**^	**Factor Weight**
(Constant)	-106.474	26.713		-3.986	0.00		
FACTOR 9-Rational entrepreneur	8.856	2.005	0.385	4.416	0.00	0.15	26%
FACTOR 6-Social-perfectionist entrepreneur	5.792	1.523	0.349	3.804	0.00	0.12	22%
FACTOR 2-Intuitive entrepreneur	8.47	2.799	0.274	3.026	0.00	0.08	13%
FACTOR 1-Entrepreneur by nature	5.238	1.848	0.270	2.834	0.01	0.07	13%
FACTOR 10-Adventurous entrepreneur	5.792	1.848	0.260	3.134	0.00	0.07	12%
FACTOR 4-Educated entrepreneur	2.892	1.475	0.170	1.961	0.06	0.03	5%
FACTOR 8-Self- confident entrepreneur	3.318	1.749	0.170	1.897	0.06	0.03	5%
FACTOR 3-Group influence entrepreneur	2.124	1.711	0.112	1.242	0.22	0.01	2%
FACTOR 5-Social influence entrepreneur	-2.199	2.185	0.090	-1.006	0.32	0.01	1%
FACTOR 7-Optimistic entrepreneur	0.613	1.954	0.028	0.314	0.76	0.00	0%
					**Sum:**	0.56	100%

b Dependent variable: Fulfilment of expectations (0 = "It did not go ahead" to 200 "It exceeded what was expected by 100%")

Source: own elaboration, 2022

## Discussion

In this study, the Exploratory Factorial Analysis (EFA) was applied to discover factors that reveal the degree of fulfilment of expectations for entrepreneurs from business incubator programmes in Madrid. Similar studies on business incubators and their impact on entrepreneurial intentions, behaviour, participation outcomes, and expectations by using Exploratory Factorial Analysis can be found in Ip et al. (2017); Ibrahim et al. (2017a); Koe (2016); Siddiqui et al. (2021) and Langkamp and Lane (2012).

The EFA analysis used for this study of expectations of fulfilment for entrepreneurs from business incubator programmes discovered 10 factors. These factors were based on 32 variables or scale items from the 7 study variables related to hard skills, soft skills, attitudes, social influence, behaviour, organisational legitimacy and business incubator programmes, as important indicators for continuation of successful entrepreneurship. The Multiple Linear Regression analysis was applied to measure individual variables of significance, making it possible to create an acceptable indicator for fulfilment of expectations for entrepreneurs who graduated from the business incubator programme.

In this research, the first factor of importance was found in Factor 9 “Rational entrepreneur” with 26% of the factor weight, where variables were related to rational and effective decision-making process and leadership. Also, other studies indicate that an individual’s perception of rational self-efficacy has a strong influence on how this person will act and how the available knowledge and skills will be utilised. Thus, there is a significant correlation between self-efficacy and entrepreneurial intention (Kristiansen & Indarti, 2004; Kristiansen et al., 2003).

Our results show a second important factor, Factor 6 “Social-perfectionist entrepreneur” with 22% of the factor weight, where variables were linked with avoidance of failure and doing everything in a correct manner, and approval of friends or family for being an entrepreneur. Comparably, some authors such as Van Gelderen et al. (2015), indicate that self-control is neither a direct predictor of whether entrepreneurial action will be taken, nor does it correlate significantly with intention strength, and it has no motivating properties by itself; however, self-control does help bring intentions to fruition. A principal role of self-control is to counter demands on volitional capacity: a person exercising strong self-control is less likely to be adversely affected by action doubt, action fear, and action aversion.

Thus, factor 2 “Intuitive entrepreneur” is the following, with 13% of the factor weight. The research variables of this factor are connected with feeling and seeing new opportunities for the ideas, markets and products. Also, Professor Cardon (2008) expressed positive impact of intuition for entrepreneurs who rely on it for seeing new business opportunities and making different decisions related to entrepreneurship.

The following Factor 1 is “Entrepreneur by nature”, where the weight is 13%. Within this factor naming, the authors include variables related to the idea that, the creation of their own company is always on their mind and entrepreneurship in general is part of their identity. Likewise, studies conducted by Cardon et al. (2005); Cardon (2008); and Cui and Bell (2022) indicate that having passion for entrepreneurship leads to accomplishment of business ideas.

Consequently, with 12% of factor weight appears Factor 10, which is named “Adventurous entrepreneur” and consist only of one variable, where risk taking regardless of results plays an important role. Similarly, Baron (2008) indicates that entrepreneurs equipped with a broader range of functional “tools” show an increased capacity for responding effectively to rapidly changing conditions in dynamic environments. Also, Forgas and George (2001) express that making decisions effectively is a crucial task in many settings, but it takes on added importance under the conditions of high uncertainty, unpredictability, and intense time pressure that entrepreneurs frequently face.

The next Factor 4 “Educated entrepreneur” has 5% weight and is sixth on the discovered importance list. This factor consists of variables that express the value of education and training for starting entrepreneurship. In this context, there are controversial studies in relation to this factor. On the one hand, authors like Souitaris et al. (2006), affirm the importance of academic education and training from specific entrepreneurship programmes and its positive impact. And on the other hand, entrepreneurship education does not directly influence entrepreneurial behaviour, but entrepreneurship education plays a significant role in business creation if students are motivated to be entrepreneurial (Lechuga et al., 2022), or that entrepreneurship education will not positively influence entrepreneurial behaviour if the entrepreneurial decision has not been made (Liñan & Fayolle, 2015).

Therefore, the following Factor 8 “Self- confident entrepreneur” with the factor weight of 5% is based on variables, where entrepreneurs have self-confidence in group leadership and market opportunities. Although, studies done by Islam et al. (2011) and Yusuf (1995), indicate that personal qualities and traits, such as self-confidence and perseverance, have importance in developing and managing the person's own business company.

Succeeding, Factor 3 “Group influence entrepreneur” (2% weight) and Factor 5 “Social influence entrepreneur” (1% weight), both factors give a similarly vital role of any external group, such as friends, family members, other business incubator members, who encourage the persons to participate in a programme or start their own company. Thus, our discovered results reveal that these factors do not play a significant role in the fulfilment of expectations for entrepreneurs from business incubator programmes. On the other hand, various studies point out that social networks are the source of many important resources for entrepreneurs—financial, human, and informational (Ozgen & Baron, 2007; Walter et al., 2006). In this context, we could interpret that those other studies indicate that social networks or “networking” is crucial to having and managing business in later stages, but our study indicates less importance in the first stage, regarding encouragement for the creation of the person's own company.

Ultimately, Factor 7, which in this research paper is named “Optimistic entrepreneur”, has obtained 0% factor weight by applying the Multiple Linear Regression analysis. This factor contains variables that are related to the opportunistic view when facing business failures. In this sense, it should be taken into account that these variables were based on studies among entrepreneurs who recently graduated from the business incubator programme and did not have many businesses failure experiences yet. Thus, author Baron (2008) states, those entrepreneurs who express a high degree of positive emotion concerning their ideas and new ventures may be more effective in generating similar positive reactions in investors, customers, potential employees, and others. Given that obtaining essential financial and human resources often involves persuading others of the value or potential of a new venture, a positive effect may contribute to the entrepreneurs’ success in their efforts to secure such resources. Also, Souitaris et al. (2006) express that optimism and learning from failures leads to an entrepreneurial opportunity.

## Conclusions

Summarising, the main results suggest that for most of the graduates from the business incubator programme, which is 90% of the total study participants in the Autonomous Community of Madrid, the outcome was beneficial for continuing to manage their business company. Consequently, it shows that academic and/or practical training programmes, apart from the university courses, play a vital role in the company´s management at the beginning or during its lifecycle for obtaining new insights and practices from educators and other associate companies in the incubator programme. Thus, in the “lifelong learning era”, learning takes place across a number of different “venues” and involves mixed-age groups in different constellations (UNESCO, 2022).

Moreover, other results of this paper indicate that ten discovered mixed factors based on 32 variables or scale items from the 7 study variables related to hard skills, soft skills, attitudes, social influence, behaviour, organisational legitimacy, and the business incubator programme lead to fulfilment of expectations for entrepreneurs graduating from the business incubator programme. Using the Multiple Linear Regression analysis, these ten factors were ordered depending on their statistical significance. It should be noticed that the factor named "Rational" is one of the key aspects to success, followed by the "Social—perfectionist entrepreneur", “Intuitive entrepreneur”, “Entrepreneur by nature” and “Adventurous entrepreneur” factors. All five of the most statistically significant factors are weighted between 12 and 26%, and the majority consist of variables that are related to behaviour and attitude. Afterwards, come factors with a weight level between 0 and 5%, which are “Self-confident entrepreneur”, “Group influence entrepreneur”, “Social influence entrepreneur” and “Optimistic entrepreneur”. These last five factors represent a mix of variables which, in a greater part, contain hard and soft skills. In this context, as general conclusion, the authors can assume that participation in business incubator programmes is fruitful for creating adequate entrepreneurial attitude, changing the behaviour toward successful management of the company and for training different hard skills, such as knowledge in how to use the latest IT systems, appliance of managerial techniques and other skills, which could be obtained through official training. On the other hand, the study shows that innate or soft skills also play some role for fulfilling expectations for developing a successful company.

Consequently, this article intends to give some *theoretical and practical contributions*. From *the theoretical point of view*, this study expands theoretical models, such as Entrepreneurial Event Model (Shapero & Sokol, 1982) and the Theory of Planned Behaviour (Ajzen, 1991), by adding new study variables for investigating the fulfilment of expectations of the business incubator programme graduates. Moreover, this study concentrates on the entrepreneurs in Spain, thus closing the theoretical gap of the Spanish entrepreneurs who graduated from the business incubators in relation to these models and study objectives. Also, this study adds some insights from the methodological point of view, by use of the Exploratory Factorial Analysis (EFA) for discovering new factors that could turn into indicators. As the last theoretical contribution, it could be added that this study is applied in 2022 and the experiences during the last few years, which were affected by the sanitary pandemic caused by the Covid-19 virus, where the study results could be influenced by this exogenous obstacle.

From *the practical point of view* and on the basis of the results obtained, the authors can recommend that obligatory life-long learning business incubator programmes should be implemented and encouraged for all kinds of entrepreneurs to train in new skills, knowledge, insights, experiences, and change of behaviours and attitudes of different aspects needed for successful company leadership and management. These training programmes should use the latest technological advances, such as gaming, virtual worlds, augmented reality, for simulating different business situations. Also, business incubator mobile learning (m-learning) platforms should be implemented to achieve more personal and effective training, and performance evaluation. Thus, the authors could suggest some *social implications* as well, such as integrating retired entrepreneurs as tutors, who could share their business and life experiences and knowledge. Also, create and integrate programmes, which teach and train on how to consume less energy for all the processes of the product/service creation and overall management of the company. Finally, in the incubator business programme, the mindset of the inclusivity policies should be included.

## Limitations and future research

This study has several limitations, which must be pointed out for understanding the context of the results obtained and providing the possibility for future research. On one hand, the research is based on the limited sample size of the 100 entrepreneurs in the study who recently graduated from the business incubators of the Autonomous Community of Madrid. The limited response is due to the three main reasons, already explained in the methodology. Firstly, the respondents' decline to participate in the survey even after a follow-up email reminder and a phone call; secondly, non-active email address from the list that was accessible to the authors and thirdly; the lack of motivation to participate in the survey. On the other hand, the factors discovered could be analysed by including exogenous variables, affected at the moment of the study, such as impact of the Covid-19 sanitary crisis, the war in Ukraine, global logistic problems, tax rate politics, technological advances, among others. Also, the study could incorporate other variables based on the academic literature, for the factor exploration on the expectation of fulfilment of entrepreneurs graduated from business incubator programme. Finally, newly obtained factor naming could include some biases, as it is based on the subjective authors´ interpretations of the non-numerical content factor (analysed variables). Thus, as it is also explained in the methodology, the factor naming bias is one of the Exploratory Factorial Analysis (EFA) technique´s steps.

In order to address the abovementioned limitations, future studies could include a larger number of business incubator graduates from other Autonomous Communities in Spain and compare results with the graduates from other countries. Furthermore, by using Structural Equation Modelling (SEM), the relation influences among newly obtained factors could be studied and exogenous factors could be included related to the country´s present economic situation and business success environment.
